# Fibrinolysis in patients with a mild-to-moderate bleeding tendency of unknown cause

**DOI:** 10.1007/s00277-016-2893-6

**Published:** 2016-12-26

**Authors:** Johanna Gebhart, Sylvia Kepa, Stefanie Hofer, Silvia Koder, Alexandra Kaider, Alisa S. Wolberg, Helmuth Haslacher, Peter Quehenberger, Ernst Eigenbauer, Simon Panzer, Christine Mannhalter, Ingrid Pabinger

**Affiliations:** 1 Department of Medicine I, Clinical Division of Hematology and Hemostaseology, Medical University Vienna, Waehringer Guertel 18-20, 1090 Vienna, Austria; 2 Center for Medical Statistics, Informatics and Intelligent Systems, Medical University Vienna, Vienna, Austria; 3 Department of Pathology and Laboratory Medicine, University of North Carolina, Chapel Hill, NC 27599-7525 USA; 4 Department of Laboratory Medicine, Division of Medical and Chemical Laboratory Diagnostics, Medical University Vienna, Vienna, Austria; 5 IT-Systems and Communications, Medical University Vienna, Vienna, Austria; 6 Department of Blood Group Serology and Transfusion Medicine, Medical University Vienna, Vienna, Austria; 7 Department of Laboratory Medicine, Division of Molecular Biology, Medical University Vienna, Vienna, Austria

**Keywords:** Bleeding of unknown cause, Hyperfibrinolysis, Plasminogen activator inhibitor-1 deficiency, Tissue plasminogen activator, Thrombin activatable fibrinolysis inhibitor, Alpha2-antiplasmin

## Abstract

In more than 50% of patients with a mild-to-moderate bleeding tendency, no underlying cause can be identified (bleeding of unknown cause, BUC). Data on parameters of fibrinolysis in BUC are scarce in the literature and reveal discrepant results. It was the aim of this study to investigate increased fibrinolysis as a possible mechanism of BUC. We included 270 patients (227 females, median age 44 years, 25–75^th^ percentile 32–58) with BUC and 98 healthy controls (65 females, median age 47 years, 25–75^th^percentile 39–55). Tissue plasminogen activator (tPA-) antigen and activity, plasminogen activator inhibitor type-1 (PAI-1), tPA-PAI-1 complexes, thrombin activatable fibrinolysis inhibitor (TAFI), α2-antiplasmin, and D-dimer were determined. While PAI-1 deficiency was equally frequent in patients with BUC and controls (91/270, 34%, and 33/98, 34%, *p* = 0.996), tPA activity levels were more often above the detection limit in patients than in controls (103/213, 48%, and 23/98, 23%, *p* < 0.0001). We found lower levels of tPA-PAI-1 complexes (6.86 (3.99–10.00) and 9.11 (7.17–13.12), *p* < 0.001) and higher activity of TAFI (18.61 (15.80–22.58) and 17.03 (14.02–20.02), *p* < 0.001) and α2-antiplasmin (102 (94–109) and 98 (90–106], *p* = 0.003) in patients compared to controls. Detectable tPA activity (OR 3.02, 95%CI 1.75–5.23, *p* < 0.0001), higher levels of TAFI (OR 2.57, 95%CI 1.48–4.46, *p* = 0.0008) and α2-antiplasmin (OR 1.03, 95%CI 1.01–1.05, *p* = 0.011), and lower levels of tPA-PAI-1 complexes (OR 0.90, 95%CI 0.86–0.95, *p* < 0.0001) were independently associated with BUC in sex-adjusted logistic regression analyses. We conclude that the fibrinolytic system can play an etiological role for bleeding in patients with BUC.

## Introduction

In the majority of adult patients with a mild-to-moderate bleeding tendency, no diagnosis can be established and the cause for the bleeding symptoms remains elusive [1]. This lack of a diagnosis subsequently hinders targeted therapy and causes high psychological strain in affected individuals and treating physicians, especially in situations with increased bleeding risk, such as surgery.

Fibrinolysis is a strictly regulated process modulated by activators and inhibitors. Hyperfibrinolytic conditions associated with bleeding tendencies of variable severity have been found in hereditary deficiencies of anti-fibrinolytic factors, such as α2-antiplasmin deficiency [2, 3] and deficiency of the plasminogen activator inhibitor type-1 (PAI-1) [4–8]. Although hyperfibrinolysis has been discussed as a possible cause for mild-to-moderate bleeding tendencies of unknown cause, the assessment of fibrinolysis is not included in the routine coagulation work-up of patients with mild-to-moderate bleeding tendencies.

Parameters of fibrinolysis have been inconsistently investigated in few cohorts with a bleeding tendency and revealed conflicting results [9–13]. Even the analysis of the same parameter in similar cohorts (e.g. patients with menorrhagia) revealed discrepancies [13, 14].

Our study aimed to investigate fibrinolysis in a cohort of patients with a mild-to-moderate bleeding tendency in whom no diagnosis could be established despite thorough investigation of hemostatic parameters. We assessed a panel of factors involved in the fibrinolytic process to determine the contribution of fibrinolysis to bleeding tendencies of unknown cause (BUC). Results in patients were compared to those of healthy controls.

## Patients and methods

### Patients

All patients had symptoms of a mild-to-moderate bleeding tendency that were recorded within the standardized questionnaire [15], encompassing skin manifestations such as hematoma or easy bruising, increased postsurgical bleeding, prolonged bleeding after tooth extraction, small wound bleeding, epistaxis, oral mucosal bleeding, gastrointestinal bleeding (not caused by pathologies in the GI-tract), spontaneous muscle or joint bleeding, and heavy menstrual bleeding and increased postpartum bleeding in females. For the current analysis, we selected patients with a mild-to-moderate bleeding tendency in whom no diagnosis could be established from two different bleeding cohorts: the Vienna Bleeding Study (VIBS) and the Vienna Bleeding Biobank (VIBB). Both studies were conducted at the outpatient department of the Division of Hematology and Hemostaseology, Medical University Vienna, Austria and were approved by the Ethics Committee of the Medical University Vienna. All procedures were in accordance with the ethical standards of the committee on human experimentation (institutional and national) and with the Helsinki Declaration of 1975, as revised in 2008. All patients and controls gave their written informed consent.

For VIBS, patient recruitment started in November 2006 and was ongoing until March 2008. Patients who had been investigated at our center in the years 2000–2004 because of a mild-to-moderate bleeding tendency and in whom no diagnosis that would explain the bleeding tendency was established were asked to participate in the study [16, 17]. The second cohort (VIBB) started in 2009 and included all patients with a mild-to-moderate bleeding tendency who were referred to the outpatient department for further investigations. A cohort of 98 unrelated subjects without a history of bleeding and/or thromboembolic events was recruited from hospital personnel, their friends, and relatives.

Patients and controls underwent a structured interview on their medical history in general and their personal and familial bleeding history in particular upon study inclusion.

Exclusion criteria that applied for patients and controls were antithrombotic and/or anti-inflammatory therapy, impaired liver function (prothrombin time <75% of normal due to a decrease in vitamin K dependent clotting factors), severe renal insufficiency, and/or thrombocytopenia (<100 × 10^9^/L). Pregnant women, subjects with active malignancy, those with surgery or delivery within 6 weeks, bacterial infection within 2 weeks prior to recruitment, or acute phase reaction at recruitment were not included. Gastrointestinal (GI) bleeds due to pathologies in the GI tract or intracerebral bleeding due to aneurysm were not regarded as spontaneous bleeding.

### Selection of patients with bleeding of unknown cause (BUC)

Patients with vWF antigen and/or activity levels of ≤50%; pathologies in the assessment of platelet function by light transmission aggregometry and/or glycoprotein expression by flow cytometry; deficiencies of the clotting factors VIII, IX, or XIII ≤50%; or the diagnosis of any other known inherited or acquired coagulation disorder were not included in this analysis. In the VIBS, as previously described, 135 patients were screened for eligibility [17]. Of them, 101 patients fulfilled the inclusion criteria and were enrolled in the study. Finally, 74 patients (73%), in whom no diagnosis according to the criteria could be established and the assessed parameters of fibrinolysis were available, were included in this data analysis. Until November 2013, 277 patients were included in the VIBB. No bleeding disorder could be found in 196 patients (71%). Thus, 270 patients with BUC were identified in the two-patient cohorts with mild-to-moderate bleeding tendencies. In the present case-control study, results were compared to those of 98 unrelated healthy controls.

### Evaluation of the bleeding severity in patients with BUC

A standardized questionnaire, previously described [15], was completed by a trained physician within a structured patient interview. The recorded bleeding symptoms were epistaxis, hematoma/easy bruising, small wound bleeding, oral mucosal and/or gingival bleeding, GI bleeding, postpartum bleeding, muscle and/or joint bleeding, bleeding after tooth extraction, postsurgical bleeding, and menorrhagia. All bleeding symptoms were scored according to their most severe occurrence. The applied bleeding score quantifies the individual bleeding tendency on a scale from 0 to 30 points.

### Sampling

Blood samples were drawn with a 21-gauge butterfly needle (Greiner Bio-One, Kremsmuenster, Austria) by antecubital venipuncture into a Vacuette tube (Greiner Bio-One, Kremsmuenster, Austria) containing trisodium citrate (nine parts of whole blood, one part of trisodium citrate 3.8%). The tubes were transferred to the MedUni Wien Biobank, a centralized sample handling and storage facility (www.biobank.at). Hence, all further processing steps occurred according to the standard operating procedures in an ISO 9001:2008 certified environment. In detail, platelet poor plasma was prepared by centrifugation at 2000*g* for 15 min at 15 °C (Hettich Rotanta 460 Robotic, Tuttlingen, Germany) and as a second step at 18,000*g* for 2 min (Eppendorf 5417R, Hamburg, Germany) and stored at <−70 °C.

### Assessment of hyperfibrinolysis

The following parameters were investigated in patients and controls: tPA antigen (U/mL) and activity (U/mL), PAI-1 antigen (U/mL), tPA-PAI-1 complexes (ng/mL), TAFI (ng/mL), α2-antiplasmin (%), fibrinogen Clauss (mg/dL), and D-dimer (μg/mL).

Tissue plasminogen activator (tPA) antigen and activity were assessed using a sandwich ELISA (tPA Actibind ELISA, Technoclone, Vienna) in which tPA is bound with a catching antibody that is not interfering with its functional activity. Bound tPA antigen is quantified with a peroxidase-labeled monoclonal antibody (mAB). tPA activity was determined using plasminogen, cyanogen bromide fragments of fibrinogen, and a plasmin substrate.

The active PAI-1 antigen (U/mL) was measured using a sandwich ELISA (PAI-1 Actibind ELISA, Technoclone, Vienna, Austria).

tPA-PAI-1 complexes were determined by ELISA (tPA-PAI-1 Complex ELISA Kit, Technoclone, Vienna, Austria) based on catching the complexes with a mAB directed against tPA. For quantification, a peroxidase-labeled mAB against PAI-1 was used.

The measurement of TAFI was based on a chromogenic assay (Stachrom TAFI, Asnières sur Seine, France) in which TAFI is first activated to TAFIa by a human-thrombin-rabbit-thrombomodulin complex and then quantified after two hydrolysation steps by measurement of discoloration at 405 nm.

The activity of α2-antiplasmin was determined by the chromogenic STA Stachrom antiplasmin assay (Diagnostic Stago, Asnieres, France). Measurement was performed on a STA-R evolution (Diagnostic Stago, Asnieres, France) at the Clinical Department of Laboratory Medicine.

Fibrinogen was quantitatively measured by the Clauss-method at the Clinical Department of Laboratory Medicine using an automated assay (STA Fibrinogen, Roche Diagnostics and Diagnostica Stago, Asnières sur Seine, France). This functional assay is based on clotting of citrated plasma in presence of an excess of thrombin, which is inversely proportional to the fibrinogen concentration. The reagent contains a heparin inhibitor. Tests were performed on the STA-R (Diagnostica Stago, Asnières sur Seine, France).

The fibrinogen degradation product D-dimer was determined by an ELISA (Asserachrom D-Di, Diagnostica Stago, Asnières sur Seine, France). Binding to a mouse-mAB is used to immobilize D-dimer. A peroxidase-labeled rabbit mAB against human fragment D was used for quantification.

### Statistics

All variables representing fibrinolysis parameters are described by their median (quartile) values. To achieve normal distribution in case of right-skewed variables, log2-transformed values were used for all statistical analyses. Group comparisons with respect to the continuous variables were performed using the two-sample *t* test. The variable tPA activity was compared using the non-parametric Wilcoxon rank sum test. The Wilcoxon rank sum test was also used to compare the assessed parameters of fibrinolysis between patients with or without specific bleeding symptoms. No correction for multiplicity was applied due to the exploratory character of this study.

Due to the high proportion of tPA activity values close to zero and below the sensitivity of the assay, tPA activity was considered as a binary variable (0 U/mL vs >0 U/mL) within the regression models. Univariate logistic regression models as well as sex-adjusted logistic regression models were performed to evaluate the strength of the fibrinolysis variables to discriminate between patients and controls. Results of the logistic regression models are given by the estimated odds ratios (OR) with 95% confidence intervals (CI) and *p* values. *P* values <0.05 were considered statistically significant.

## Results

### Characteristics of patients and controls

Clinical and laboratory characteristics of patients with BUC and controls are shown in Table 1. There was a female predominance in our group of patients with BUC. Blood groups were available for 245 patients (90.7%) and 97 healthy controls (99%). Blood group O occurred with an equal frequency in patients (42.4%) and controls (44.3%). There was also no difference in age or body mass index (BMI) between the groups.

**Table 1:** Clinical and laboratory characteristics of patients and controls

	Patients (*n* = 270)	Controls (*n* = 98)
Female, n (%)*	227 (84.1)	65 (66.3)
BG 0, n (%)^a^	104 (42.4)	43 (44.3)
Family history, n (%)^b^	75 (34.7)	
	Median (25–75 percentile)	Median (25–75 percentile)
Age (years)	44 (32–58)	47 (39–55)
BMI (kg/m^2^)	23.7 (21.3–27.1)	23.8 (21.5–25.9)
Hemoglobin (g/dL)*	13.7 (12.8–14.5)	13.9 (13.2–14.9)
Platelet count (10^9^/L)	244 (216–278)	248 (220–286)
PT (%)	116 (106–126)	114 (103–125)
APTT (sec)	35.2 (33.2–38.0)	35.5 (33.3–37.4)
Bleeding score	4 (3–6)	–

*BG* blood group, *BMI* body mass index, *PT* prothrombin time, *APTT* activated partial thromboplastin time

*Statistically significant difference between the groups

^a^Available of 245 patients (90.7%) and 97 healthy controls (99.0%)

^b^Family history of bleeding available of 216 patients (80.0%)

Interestingly, patients had lower levels of hemoglobin. As this difference could be due to the female predominance in the patient group, we adjusted for sex, and indeed, then the difference disappeared (*p* = 0.71) (data not shown). There was no difference in platelet count or global clotting tests such as prothrombin time (PT, %) and activated partial thromboplastin time (APTT, seconds) between patients with BUC and controls.

Table 2 depicts the occurrence of each bleeding symptom and the proportion of manifestations requiring medical intervention at least once.

**Table 2:** Occurrence of bleeding symptoms and requirement for medical interventions

	Frequency			Medical intervention required	
	*N*	*n*	%	*n*	%
Hematoma/easy bruising	270	176	65.2	12	6.8
Increased postsurgical bleeding^a^	251	145	57.8	79	54.5
Abnormal bleeding after tooth extraction^a^	239	88	36.8	18	20.5
Increased bleeding from small wounds	270	87	32.2	0	0
Epistaxis	270	73	27.0	25	34.2
Increased oral mucosal bleeding	270	32	11.9	0	0
Gastrointestinal bleeding	270	25	9.3	6	24.0
Muscle or joint bleeding	270	4	1.5	0	0
Menorrhagia^b^	227	147	64.7	90	61.2
Abnormal postpartum bleeding^a,b^	157	53	33.8	25	47.2

^a^Patients exposed to surgery or tooth extraction or delivery

^b^All women

### Parameters of fibrinolysis in patients and controls

Table 3 shows the results for the fibrinolytic parameters in patients with BUC in comparison to healthy controls. Patients had significantly higher tPA activity than healthy controls. tPA activity was measurable (>0 U/mL) more frequently in patients than in healthy controls. Levels of tPA-PAI-1 complexes were lower in the group of patients with BUC. Levels of fibrinolysis-inhibitors TAFI and α2-antiplasmin were increased in the group of patients and also fibrinogen was higher in patients with BUC in comparison to healthy controls.

**Table 3:** Parameters of fibrinolysis in patients with BUC and controls

	Patients	Controls	*p*
	Median (25–75 percentile)	Median (25–75 percentile)	
tPA antigen (U/mL)^a,^*	1.75 (1.10–2.65)	2.26 (1.34–3.76)	0.096
tPA activity (U/mL)^a^	0.00 (0.00–0.13)	0.00 (0.00–0.00)	<0.001
PAI-1 antigen (U/mL)*	1.9 (0.7–5.1)	2.5 (0.5–6.5)	0.809
tPA-PAI-1 complex (ng/mL)^a^	6.86 (3.99–10.00)	9.11 (7.17–13.12)	<0.001
TAFI (ng/mL)^b,^*	18.61 (15.80–22.58)	17.03 (14.02–20.02)	<0.001
α2-antiplasmin (%)	102 (94–109)	98 (90–106)	0.003
Fibrinogen (mg/dL)	324 (281–375)	312 (261–343)	0.003
D-dimer (μg/mL)^c,^*	0.28 (0.18–0.47)	0.28 (0.19–0.40)	0.194
	*n* (%)	*n* (%)	*p*
PAI-1 antigen <1 U/mL	91 (34)	33 (34)	0.996
tPA activity >0 U/mL^a^	103 (48)	23 (23)	<0.0001

*tPA* tissue plasminogen activator, *PAI-1* plasminogen activator inhibitor-1, *TAFI* thrombin activatable fibrinolysis inhibitor

*log2 transformed variables used for the comparison between the groups

^a^Available of 213 (78.9%) patients

^b^Available of 263 (97.4%) patients

^c^Available of 262 (97.0%) patients

In logistic regression analyses, detectable tPA activity >0 U/mL, low tPA-PAI-1 complexes, increased levels of TAFI, α2-antiplasmin, and fibrinogen remained significantly associated with BUC also after adjustment for sex (Table 4).

**Table 4:** Sex-adjusted logistic regression analyses of parameters of fibrinolysis

	OR	95%CI	*p*
tPA antigen*	0.87	0.72–1.06	0.16
tPA activity >0 U/mL	3.02	1.75–5.23	<0.0001
PAI-1 antigen*	1.06	0.95–1.19	0.30
tPA-PAI-1 complex (ng/mL)	0.90	0.86–0.95	<0.0001
TAFI*	2.57	1.48–4.46	0.0008
α2-antiplasmin (%)	1.03	1.01–1.05	0.011
Fibrinogen (10 mg/dL)	1.04	1.00–1.08	0.030
D-dimer*	1.05	0.83–1.34	0.68

*tPA* tissue plasminogen activator, *PAI-1* plasminogen activator inhibitor-1, *TAFI* thrombin activatable fibrinolysis inhibitor

*log2-transformed

We analyzed the presence of PAI-1 deficiency defined as plasma levels of PAI-1 antigen <1 U/mL, a cut-off level also used by Agren et al. [9, 10], as possible cause for BUC. In our population, PAI-1 deficiency occurred with equal frequency in patients and controls (Table 3). Activity of α2-antiplasmin below normal (<75%) was found in five study participants, two patients, and three healthy controls. None of the patients or healthy controls had levels of α2-antiplasmin below 60%.

There was no specific pattern of altered fibrinolysis that would correlate with any of the bleeding symptoms. Patients with skin manifestations had higher levels (median (IQR)) of fibrinogen (330 (294–377) and 307 (266–364), *p* = 0.017) and D-dimer (0.32 (0.20–0.48) and 0.23 (0.16–0.35), *p* = 0.003) than patients without skin manifestations. Likewise, levels of TAFI (19.86 (16.34–25.77) and 18.42 (15.49–21.88), *p* = 0.023) and D-dimer (0.36 (0.22–0.62) and 0.25 (0.17–0.41), *p* < 0.001) were increased in patients with prolonged bleeding from small wounds compared to those without; levels of TAFI were also increased in patients with pathological postsurgical bleeding compared to those with a normal postsurgical course (19.60 (16.34–23.04) and 18.23 (15.20–22.58), *p* = 0.043). There was no difference in the assessed fibrinolytic factors between patients with or without increased oral mucosal bleeding, GI bleeding, or prolonged bleeding after tooth extraction. In females, we found no difference in the parameters of fibrinolysis between patients with or without heavy menstrual bleeding. Patients with a history of increased postpartum bleeding had slightly lower levels of D-dimer compared to those with a normal postpartum course (0.30 (0.20–0.47) and 0.37 (0.25–0.63), *p* = 0.047).

## Discussion

We investigated a broad panel of parameters with pro- and anti-fibrinolytic capacity to get a clearer picture about the involvement of fibrinolysis in BUC. The results indicate alterations in the fibrinolytic process as a cause for increased bleeding in patients with BUC. Up to now, parameters involved in the fibrinolytic process were examined only in small patient cohorts with a bleeding tendency, and the results were controversial [10–14, 18].

We found significantly increased tPA activity, lower levels of tPA-PAI-1 complexes, and slightly but significantly increased levels of TAFI, α2-antiplasmin, and fibrinogen in our patients with BUC compared to the group of healthy controls. These factors were also independently associated with BUC in sex- adjusted logistic regression analyses.

Our findings of increased tPA activity and lower levels of tPA-PAI-1 complexes may indicate an impaired inhibition of tPA by PAI-1. This could result from a functional defect of one of the ligands, as there was no difference in PAI-1 activity or tPA antigen levels between patients and controls in our cohort. This impaired inhibition of tPA could result in higher fibrinolytic activity and thus be responsible for the bleeding tendency in our patients with BUC. To the best of our knowledge, we are the first to investigate tPA activity in patients with a bleeding tendency, as previous studies only investigated tPA antigen levels [10, 13, 14]. Likewise, quantitative measurements of tPA-PAI-1 complex levels were only performed in a small number of studies on patients with a bleeding tendency and results are inconclusive [10, 12, 18]. In line with our data, one study reported lower preoperative tPA-PAI-1 complex levels in association with increased bleeding after cardiac surgery [12].

Levels of TAFI and α2-antiplasmin and fibrinogen were slightly, but significantly increased in our patients with BUC compared to controls and remained significantly associated with BUC after adjustment for sex. This increase in anti-fibrinolytic factors is surprising and difficult to explain. Still, it was the most consistent finding when patients with a bleeding tendency were compared to control individuals. In line with our data, increased levels of TAFI and α2-antiplasmin have been reported in women with strong menstrual bleeding [14]. High TAFI levels have also been associated with a more severe bleeding phenotype in patients with hemophilia A and in patients with hereditary mucocutaneous hemorrhages [10, 11]. In the latter studies, α2-antiplasmin was not investigated. This increased anti-fibrinolytic capacity might balance the augmented profibrinolytic activity of tPA depicting the steady state in patients with BUC, which prevents spontaneous and severe bleeding episodes. Additional triggers, such as invasive procedures, minimal trauma, or minor injuries, might tip this balance towards bleeding, manifesting as a mild-to-moderate bleeding tendency.

We have previously shown that there was no difference in the overall fibrinolytic capacity, represented by the half lysis time t_50_ in the turbidimetric assessment of plasma clot lysis between a subgroup of our patients with BUC and controls. This finding is in line with the results of Wiewel-Verschueren et al. [14] but in contrast to the findings of Szczepaniak et al. [13], both studies on patients with heavy menstrual bleeding. Overall fibrinolytic capacity is determined after addition of rtPA. This profibrinolytic stimulus might mask subtle, but relevant differences of the fibrinolytic activity. Thus, the clot lysis time measured in plasma-based assays might be more an expression of a clot structure with a higher susceptibility to fibrinolysis than an indicator of the individual endogenous fibrinolytic potential.

Our study has several limitations. First, patients and controls were of comparable age, but there was a female predominance in the patient group. Still, the described differences in fibrinolysis parameters remained statistically significant after adjustment for sex. Second, the fibrinolytic activity follows a circadian variation, with peak levels of PAI-1 and tPA antigen and the lowest tPA activity in the morning [19]. We considered that and restricted the blood sampling time to 8.00–10.00 for both patients and controls. Further limitations include pre-analytical factors in the determination of parameters of fibrinolysis. TPA activity has been reported as unstable, especially in the presence of high PAI-1 levels [20]. Thus, it has been recommended to determine fibrinolytic variables, especially tPA, in samples of acidified citrated plasma (Stabilyte™) [21–24], which inhibits tPA-PAI-1 complex formation and tPA inactivation [20, 25]. However, tPA-PAI-1 complex levels in citrated plasma have been reported to be only marginally increased [23]. Strengths of our study are the large number of well-characterized patients with BUC, and the systematic investigation of multiple parameters known to regulate fibrinolysis.

In summary, we have measured a comprehensive panel of parameters to assess fibrinolysis in a large cohort of patients with a mild-to-moderate bleeding tendency of unknown cause. We found alterations in fibrinolysis parameters, indicating increased fibrinolytic activity, but also increased protection against fibrinolysis in patients with BUC in comparison to healthy controls. This observation is novel and might depict the resting state of patients at risk for fibrinolytic bleeding after hemostaseological challenges. The hypothesis of increased fibrinolysis in patients with BUC is supported by the clinical efficacy of anti-fibrinolytic agents, such as tranexamic acid, in patients with mild bleeding symptoms [26–28]. We could not confirm prior reports of PAI-1 deficiency [18] as a risk factor for BUC in our cohort. Our data warrant further studies to investigate and characterize the pathophysiological processes underlying fibrinolysis in patients with BUC.
